# Fifty-year follow-up and Strampelli anterior chamber intraocular lens

**DOI:** 10.3205/oc000028

**Published:** 2015-09-10

**Authors:** Carmen L. Oakley, Matthew A. Nigro, Brendan J. Vote

**Affiliations:** 1 Tasmanian Eye Institute, Launceston, Tasmania, Australia; 2 Launceston Eye Institute, Launceston, Tasmania, Australia

## Abstract

The Strampelli anterior chamber intraocular lens was created in 1953, and was primarily used to treat myopia and aphakia. Due to the positioning of the lens, it was associated with a number of complications, and was later modified to decrease the rate of significant complications, including endothelial cell loss.

This paper describes a 62-year-old man, who has had a Strampelli intraocular lens (IOL) in situ for 52 years, with relatively few complications. The case provides a framework for reflection on the significant advances in the development of IOLs since the Strampelli era.

The Strampelli anterior chamber intraocular lens was created in 1953, and was primarily used to treat myopia and aphakia. Due to the positioning of the lens, it was associated with a number of complications, and was later modified to decrease the rate of significant complications, including endothelial cell loss.

This paper describes a 62-year-old man, who has had a Strampelli intraocular lens (IOL) in situ for 52 years, with relatively few complications. The case provides a framework for reflection on the significant advances in the development of IOLs since the Strampelli era.

## Introduction

Created in 1953 by Italian surgeon Dr. Strampelli, the Strampelli lens revolutionized Intraocular Lens (IOL) implant positioning and furthermore became a building block for further IOL developments. Sparked by faults in the lens positioning used by Ridley, Strampelli along with Dannheim [1] and Baron believed the best anatomical space for lens manoeuvrability and surgical access was the irido-corneal angle, otherwise known as the anterior chamber angle. 

Used primarily to treat myopia and aphakia, the lens is characterized by a tripod shape, allowing for a three point fixation within the chamber angle, giving the lens a greater amount of stability, and helping facilitate iridectomy at the end of the procedure [2]. However, this lens did not come without complications. A study published by Barraquer in 1959 [3] outlined 5-year post-op patient outcomes, as a result of the Strampelli lens implantation [4]. Some of the common complications associated with the early anterior chamber intraocular lenses included reduced corneal endothelial cell count leading to corneal decompensation, glaucoma related to angle obstruction or distortion, dislocation of the implant, and sympathetic ophthalmia [5], [6].

Since the Strampelli era of anterior chamber intraocular lens design, the design has been further modified to address some of the concerns relating to corneal issues by improving the supports holding the lens in place, and therefore reducing corneal endothelial damage [5].

We report the case of a 62-year-old gentleman, who has had a Strampelli anterior chamber intraocular lens in situ for 52 years. 

## Case report

This 62-year-old gentleman suffered a penetrating eye injury by a metallic foreign body in the left eye in 1963 (at 10 years of age) and subsequently developed traumatic cataract in the left eye. Approximately three months after the initial injury he underwent extraction of the traumatic cataract, and insertion of a Strampelli anterior chamber lens with intraoperative iridectomies. 

He was seen in our clinic recently with complaints consistent with lenticular opacity in the right eye. The Strampelli IOL has been in-situ in the left eye for 52 years and apart from intermittent tenderness of the eye the patient does not report any significant issues and maintains good visual function in the left eye.

**Figure 1:**
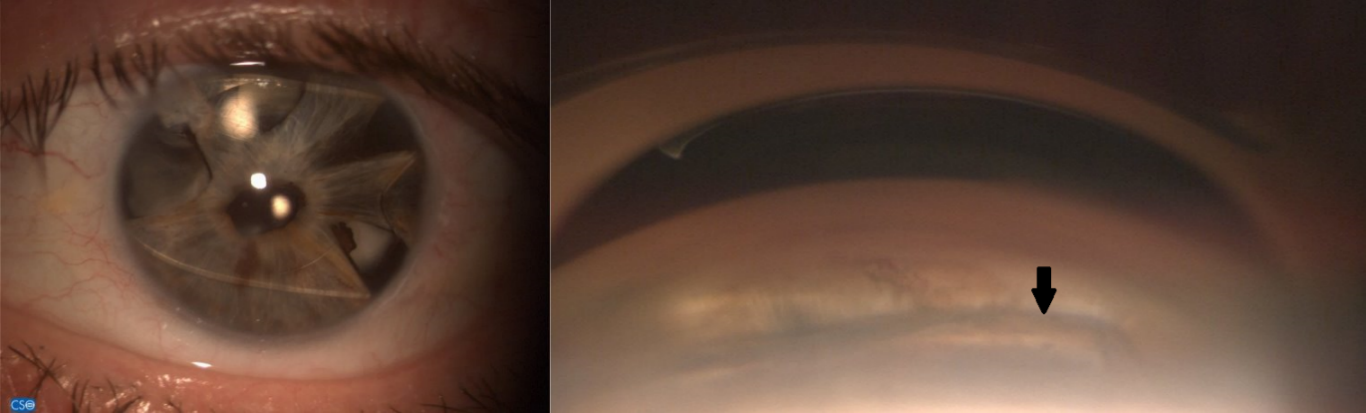
Anterior chamber photo (left) and gonioscopy photo (right), demonstrating the Strampelli lens within the anterior chamber (arrow highlighting one tail of IOL partially within iris stroma), opposite fixation (not shown) similarly posteriorly placed in iris

His best corrected visual acuity is 6/6 right eye and 6/7.5 left eye. Intraocular pressure was 14 mmHg in the right eye and 12 mmHg in the left eye. On examination the cornea was clear and anterior chamber quiet bilaterally. The strampelli lens can be seen in the anterior chamber of the left eye, along with a soemmering’s ring which can be seen through the iridectomies (see Figure 1 (Fig. 1)). The Strampelli IOL appears to be fixated posteriorly, within the iris plane on both sides rather than within the drainage angle. Examination of the right eye was unremarkable, apart from early lenticular opacity. Fundus examination was normal in both eyes. 

**Figure 2:**
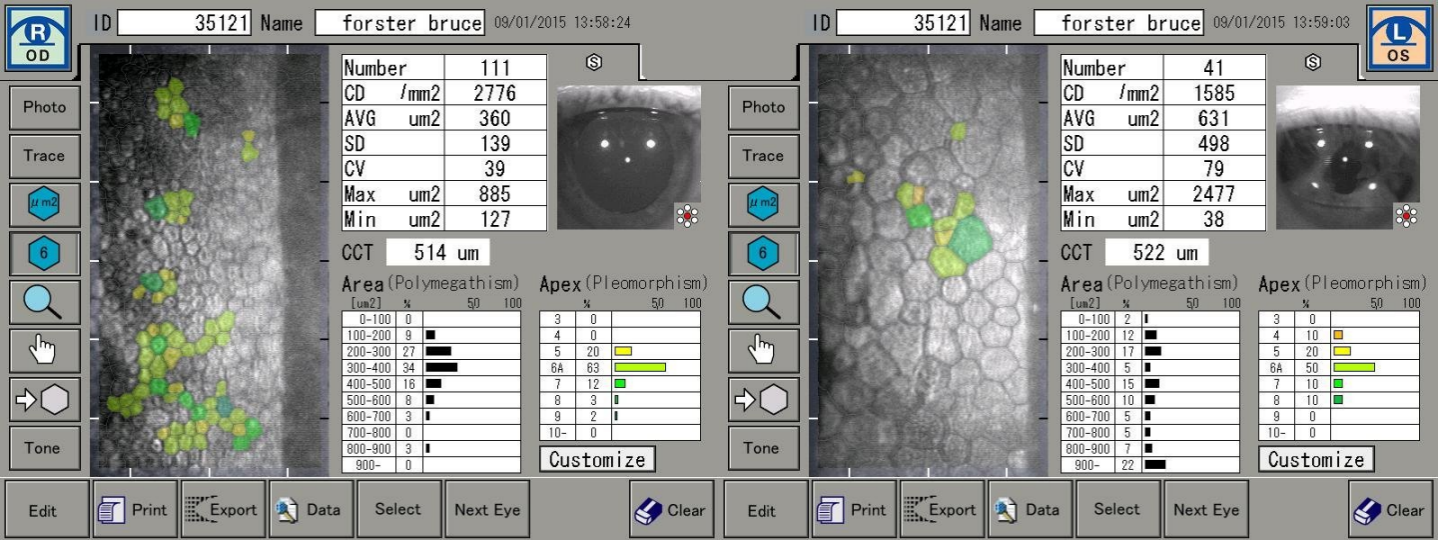
Specular microscopy images of both eyes, highlighting the much lower endothelial cell count in the left eye

Specular microscopy reveals the endothelial cell count in the right eye is 2,776 cells/mm^2^ compared to 1,585 cells/mm^2^ in the left eye (Figure 2 (Fig. 2)).

## Discussion

This case highlights that although these lenses were associated with potential complications, many patients benefitted from their use, particularly as suggested by Dr. Strampelli in the setting of unilateral aphakia unable to be managed with corneal contact lenses [7]. 

In this patient the endothelial cell count is much lower in the left eye compared with the right eye. Corneal endothelial decompensation is a known potential complication of Strampelli anterior chamber intraocular lenses [6]. A number of studies have looked into the normal rate of endothelial cell loss across a variety of age groups. It is generally accepted that the rate of endothelial cell loss is greatest from 0–20 years. In some studies the rate during this age group is estimated to be around 1.1%, with the rate settling to around 0.6% per year from the age of 18 years onwards [8], [9]. Yee et al. report the decrease in endothelial cell count to be approximately 0.3% per year from the age of 10–89 [10]. The rate of endothelial cell loss is reported to be much higher in eyes with regional trauma. Bourne et al. report the rate of endothelial cell loss in this group as high as 2.5% per year at 10 years after cataract extraction [11], however there is no data about the ongoing rate of endothelial cell loss in patients who have suffered monocular trauma early in life. 

This patient has an endothelial cell count of 2,776 in the right eye. If you apply the average endothelial cell loss of 0.3% per year from the age of 10 onwards, this would equate to roughly 3,300 endothelial cells/mm^2^ at the time of the injury. Considering the patient’s current endothelial cell count of 1,585 cells/mm^2^ in the left eye, the average endothelial cell loss is around 1% per year for the last 52 years. However, this patient has two factors which account for the increased rate of endothelial cell loss in the left eye; regional trauma and anterior chamber IOL. Since the two factors cannot be separated, it is unclear to what extent the anterior chamber IOL has contributed to the increased rate of cell loss. Additionally, the relatively increased distance between the IOL and corneal endothelium due to the positioning of the IOL’s fixation within the iris plane may have resulted in the lens having less of a detrimental effect on the corneal endothelium. 

## Conclusions

In conclusion, although the Strampelli lens has now been surpassed in technology this particular patient has benefited greatly from the use of the lens, with no morbidity currently attributable to his lens. This case highlights the significant advances made in the history of IOL development and the need to acknowledge those such as Dr. Strampelli whose shoulders we stand on. In revisiting our history, we can better look to further future developments, and long-term outcomes of prior advances are an important part of that.

## Notes

### Competing interests

The authors declare that they have no competing interests.
